# Vaginal Atrophy following Long-Term Depot Medroxyprogesterone Acetate Use: A Case Report

**DOI:** 10.1155/2013/835316

**Published:** 2013-03-06

**Authors:** Christie Walker, Shawky Z. A. Badawy

**Affiliations:** Department of Obstetrics and Gynecology, Upstate Medical University, 750 East Adams Street, Syracuse, NY 13210, USA

## Abstract

Depot medroxyprogesterone acetate (DMPA) is a commonly used form of contraception, with noncontraceptive benefits for the user. The mode of action is through the suppression of ovulation. It leads to hypoestrogenism which causes dryness of the vagina and dyspareunia. We present in this paper a patient that was very symptomatic with regard to vaginal atrophic changes determined by vaginal cytology. This side effect may become increasingly more common as we see more long-term use of DMPA.

## 1. Introduction

One of the major milestones of the 20th century was the advent of safe and reliable contraceptives for women [1]. Presently women have a multitude of options when it comes to their control of fertility, options not given to their grandmothers. However, with this new freedom comes a medical tightrope walk balancing between contraceptive benefits and their associated risks. Many younger women, who have years of contraceptive use ahead of them, prefer forms of contraception that do not require daily usage [2]; a common choice is depot medroxyprogesterone acetate (DMPA). Nearly 14% of teenagers and 10% of women in their early twenties rely on DMPA to prevent unwanted pregnancies [3]. The increasing use of DMPA has been cited as a major contributor to the declining adolescent pregnancy rates over the past 20 years [4].

DMPA is tolerated relatively well. The main reason for discontinued use is altered or unpredictable menstrual bleeding, with continuation rates at 1 year being 27%–33% [5–7]. The most common side effects associated with DMPA use are bothersome but benign and typically improve with time or upon discontinuation, including irregular bleeding, weight gain, dizziness, abdominal pain, and anxiety [7]. Occasionally, other side effects do occur and may cause significant distress to the patient. This is represented in a case report of vaginal atrophy associated with long term DMPA use. 

## 2. Case Report

Twenty-four-year-old G1P1001 single Caucasian female, who had been on DMPA for seven years, presented with complaints of dyspareunia and urinary urgency with nocturia. She stated that the dyspareunia had been present and worsening for 4 years. Dyspareunia occurred throughout intercourse, worse with rear entry, and had caused her to limit sexual activity. Her urinary complaints included burning with urination, urgency, and nocturia greater than three times per night for the last 2 years. She had previously seen a urologist and had urodynamics and cystoscopy which was negative. At that time, she received treatment with 6-dimethyl sulfoxide which showed improvement in her nocturia to one void per night. However, all other symptoms persisted unchanged. Past medical and surgical history was negative. On physical exam, vaginal atrophy was noted. Pap smear, urodynamics, urine culture, and cystoscopy were performed in the office, and all were within normal limits except for filling cystometrography values that were low. Patient was unable to tolerate potassium sensitivity testing and developed urethral bleeding after initial bladder distention. Vaginal maturation index was performed and found to be 5% in the superficial layer and 95% in intermediate and 0% in parabasal layers. Patient was informed of diagnosis of atrophic vaginitis likely secondary to long-term depo-provera use. Patient was instructed to discontinue depo-provera, given prescription to start combined oral contraceptive pills and vaginal estrogen cream. At 5-month followup, the patient reported improvement in dyspareunia, and she has some pain only with penetration. Urinary complaints were only present for first 1-2 voids after intercourse. Pelvic exam was within normal limits with no signs of vaginal atrophy. Vaginal maturation index was scheduled to be completed at 6-month follow-up visit; however, patient did not return for continued follow-up care. 

## 3. Discussion

Vaginal atrophy is characterized by vaginal burning, itching, dyspareunia with and or without decreased sexual interest. There may also be associated urinary symptoms [8]. Vaginal atrophy predominantly occurs in postmenopausal women due to a hypoestrogenic state. This may occur in the setting of DMPA use due to chronic suppression of estradiol levels.

DMPA works in a similar fashion to other progesterone only contraceptives. It causes an alteration in the endometrial lining making it inhospitable to implantation. DMPA causes a thickening of cervical mucus impeding the passage of sperm into the endometrial cavity. Ovulation does not occur with DMPA use due to suppression of gonadotropins which prevents luteinizing hormone (LH) surge and in turn prevents ovulation [4]. DMPA differs from other progesterone only contraceptives in that it does not suppress follicle stimulation hormone as strongly as LH. This results in relatively normal estradiol levels in one-third of users [4]. The remaining DMPA users will have lower estradiol levels, which can be as low as postmenopausal levels [9, 10]. Vasomotor symptoms do not typically occur with DMPA use as circulating medroxyprogesterone levels are high enough to suppress such symptoms [4]. However, with prolonged use, chronic suppression of FSH leads to diminished estradiol and progesterone production such that symptoms occur. Other effects typically seen in postmenopausal women can also occur with long-term DMPA use such as a loss in bone density [4]. Increased bone resorption is seen with DMPA use due to an overall hypoestrogenic state caused by FSH suppression. This finding led the FDA to issue a black box warning regarding loss of bone density and recommended its use to be limited to no more than 2 years, unless no other contraception was available [11]. Hypoestrogenic condition is a significant factor in causing dyspareunia as shown in this case. This is due to atrophy of vaginal epithelium and lack of secretions. Treatment for such symptoms is with discontinuation of DMPA and use of topical estrogen replacement [12]. This may improve sexual symptoms sooner than conservative management, even before vaginal estrogen levels return to normal levels.

This patient had a vaginal maturation index (VMI) performed that measured the amount of estrogen influence within her vaginal mucosa; the test confirmed atrophic vaginitis. This test is performed from a sample of cells collected from the lateral vaginal wall with light scraping. The cells are then tested for their qualitative response to estrogen [13]. The cells are categorized as parabasal, intermediate, and superficial cells with the total being equal to 100%. Normal ranges change throughout a patient's lifetime depending on their current hormone state, such as pregnancy versus postmenopausal. The VMI is of value in monitoring patient's response to hormone therapy, particularly postmenopausal response to local estrogen. Estrogen causes an increase in the superficial layers, while progestins cause an increase in the intermediate layers [14]. An overall lack of hormonal influence causes all cell layers to be close to or actually equal. This patient's response was characteristic for progesterone influence and was much more exaggerated than is usually seen in postmenopausal women. 

## 4. Conclusion

Vaginal atrophy and other postmenopausal symptoms are rare in the setting of long-term DMPA use. When the symptoms occur, it is presumed that they are reversible with discontinuation of DMPA. However in case of vaginal atrophy, exogenous estrogen may be needed to treat symptoms and hasten the return of normal sexual function. Close followup of these patients is needed to evaluate any long-term sequelae or residual dysfunction that may require additional treatment. Upon discontinuation of DMPA, the patient should be started on an alternative form of contraception if pregnancy is not desired.
